# A 178-clinical-center experiment of integrating AI solutions for lung pathology diagnosis

**DOI:** 10.1038/s41598-023-27397-7

**Published:** 2023-01-20

**Authors:** Bulat Ibragimov, Kirill Arzamasov, Bulat Maksudov, Semen Kiselev, Alexander Mongolin, Tamerlan Mustafaev, Dilyara Ibragimova, Ksenia Evteeva, Anna Andreychenko, Sergey Morozov

**Affiliations:** 1 Department of Computer Science, University of Copenhagen, Copenhagen, Denmark; 2 Research and Practical Clinical Center for Diagnostics and Telemedicine Technologies of the Moscow Healthcare Department, Moscow, Russia; 3 School of Electronic Engineering, Dublin City University, Dublin, Ireland; 4 Innopolis University, Innopolis, Russia; 5 Nova Information Management School, Universidade Nova de Lisboa, Lisbon, Portugal; 6 University Clinic Kazan State University, Kazan, Russia; 7 Osimis SA, Liege, Belgium

**Keywords:** Image processing, Machine learning

## Abstract

In 2020, an experiment testing AI solutions for lung X-ray analysis on a multi-hospital network was conducted. The multi-hospital network linked 178 Moscow state healthcare centers, where all chest X-rays from the network were redirected to a research facility, analyzed with AI, and returned to the centers. The experiment was formulated as a public competition with monetary awards for participating industrial and research teams. The task was to perform the binary detection of abnormalities from chest X-rays. For the objective real-life evaluation, no training X-rays were provided to the participants. This paper presents one of the top-performing AI frameworks from this experiment. First, the framework used two EfficientNets, histograms of gradients, Haar feature ensembles, and local binary patterns to recognize whether an input image represents an acceptable lung X-ray sample, meaning the X-ray is not grayscale inverted, is a frontal chest X-ray, and completely captures both lung fields. Second, the framework extracted the region with lung fields and then passed them to a multi-head DenseNet, where the heads recognized the patient’s gender, age and the potential presence of abnormalities, and generated the heatmap with the abnormality regions highlighted. During one month of the experiment from 11.23.2020 to 12.25.2020, 17,888 cases have been analyzed by the framework with 11,902 cases having radiological reports with the reference diagnoses that were unequivocally parsed by the experiment organizers. The performance measured in terms of the area under receiving operator curve (AUC) was 0.77. The AUC for individual diseases ranged from 0.55 for herniation to 0.90 for pneumothorax.

## Introduction

Automated diagnosis of lung diseases from chest X-rays is one of the earliest topics of interest in the field of computerized medical image analysis with the first papers published in the 60 s^1^. The automated chest X-ray analysis has evolved dramatically in the last 60 years and more than 10,000 papers can be found in Google Scholar and Pubmed using relevant combinations of keywords “lung X-ray”, “automated”, “machine learning”, “segmentation”, etc. Despite high interest in the field, only a relatively small fraction of the published algorithms are validated on external databases collected at different hospitals^2^.

The lack of comprehensive external validation compromises the practical applicability of an algorithm. The issue has received additional attention after the outbreak of COVID-19 epidemics and the rapid growth of publications on the topic in 2020 and 2021. Wynants et al.^3^ and Roberts et al.^4^ assessed the risk of bias and potential applicability of COVID-19 models using the PROBAST tool. The database composition and training–testing protocol were identified as the main sources of bias in the published models. The database composition can increase the bias of the reported results when it is unclear how the target patients were selected, or some patients were excluded from the analysis without well-justified reasons, or when the database is biased in terms of demographics. Topol^5^ has pointed out that machine learning studies are often focused on a small number of potential abnormalities, which limits the practical adoption of such studies as physicians are rarely in a position when only a few diseases are expected. To minimize the training–testing bias, the models should be tested on external databases potentially collected at different hospitals. The conclusion of Roberts et al.^4^ was that no artificial intelligence (AI) solution for COVID-19 diagnosis satisfies all the requirements for potential clinical adoption. Cohen et al.^6^ used seven publicly available databases with labeled chest X-rays and investigated how the automatic diagnostic accuracy changes when an algorithm is trained and tested on databases from the same vs different clinical sources. They observed that the accuracy measured in terms of the area under receiving operator curve (AUC) can drop from 0.90 to 0.60 for some abnormalities. Similarly, Kitamura and Deible observed an AUC drop from 0.90 to 0.59 when a pneumothorax detection algorithm trained and validated on a public database was tested on a separate private database of chest X-rays^7^.

In 2020, an experiment to link 178 clinical centers, i.e. hospitals and polyclinics, in Moscow, Russia – a region with more than 20 million inhabitants – into a single network where the X-rays and radiological reports were automatically redirected to a research facility, analyzed, and returned to the centers have been conducted^8^. The aim of this experiment was to objectively test the performance of machine learning solutions for X-ray analysis in a maximally realistic real-time environment on a megapolis-level scale. There was no restriction on the quality of the input X-ray images from the participating hospitals or the expected abnormalities. Moreover, it was expected that the participating frameworks would be able to automatically recognize if an input image does not represent a chest X-ray. The research teams from public institutions and private enterprises were invited to participate with monetary awards for the most successful teams. In this paper, we present the results of the top-performing AI-based framework.

## Results

### Experiment design

The experiment was organized by the Government of Moscow, and conducted under the technical supervision of the Research and Practical Clinical Center for Diagnostics and Telemedicine Technologies of the Moscow Health Care Department (PCCDTT) in 2020. The experiment participants were asked to develop a software solution that will be able to handle a continuous flow of X-rays images from 178 clinical centers, auto-diagnose chest abnormalities from these X-rays in a close-to-realtime fashion, and send back the results. A participating solution should operate for one month of the experiment duration. The total number of X-rays to be analyzed was not known in advance because the X-rays were acquired during the routine operation of the participating clinical centers. One of the key features of the experiment was the fact that PCCDTT did not provide any labeled chest X-rays for the algorithm training to minimize the risks of tuning the algorithms toward images from particular hospitals. At the same time, PCCDTT provided two small validation databases of 100 chest X-rays each without reference diagnoses to check if the participating algorithms satisfy the minimum performance requirements. During the experiment, the testing cases were acquired in real-time from Moscow healthcare centers and were automatically sent to the servers of participating teams. In particular, a chest X-ray was first acquired and read by radiologists from the healthcare centers following the standard workflow protocol. The X-ray and the corresponding radiologist’s diagnosis were automatically sent to PCCDTT. From PCCDTT, the X-ray was sent to the servers of all experiment participants. During a short time frame, a participating solution should return the autodiagnosis results in the PCCDTT format. These results were then compared to the radiologist’s diagnosis by PCCDTT. The workflow of the participating healthcare centers, therefore, remained uninterrupted and unaffected. In total, 178 centers contributed to the data, with 126 and 48 centers specializing in outpatient and inpatient care. The experiment was initiated by the Moscow Ministry of Health and Family Welfare department. This study is a part of a registered clinical trial on the use of AI technology for computer-aided diagnosis https://clinicaltrials.gov/ct2/show/NCT04489992.

The experiment task was formulated as the binary detection of lung abnormalities. Its overarching aim was to figure out if AI solutions can be used to prescreen chest X-rays, identify cases with potential abnormalities, and alert physicians about such cases. Most of the chest X-rays in the experiment were first interpreted by the attending radiologists, who wrote standardized radiological reports with the findings detected. These X-rays and the reports were automatically parsed by the natural language processing (NLP) algorithm, which concluded if the X-ray contains an abnormality according to the opinion of the attending physician^9^. At the same time, the X-rays were anonymized and transferred to the servers of each team participating in the experiment. A participating solution must label the received X-ray and send back the labeling result to the PCCDTT server in a predefined time frame.

### First external validation

Each participating team must have conducted two rounds of external validations of their solution before entering the experiment. The framework presented in this paper was externally validated using the data from two hospitals, namely the Republican Clinical Oncological Dispensary (RCOD), Kazan, and Republican Clinical Hospital (RCH), Kazan. In total, 91 chest X-rays from RCOD and 89 chest X-rays from RCH were analyzed. In terms of diagnoses, 110 chest X-rays had no lung abnormalities, while 80 chest X-rays had at least one lung abnormality including pneumothorax, pneumonia, lung nodules, etc. The images were of different quality and scanned using different imaging equipment.

The framework validation on the two external databases was performed using the metrics specified by the experiment organizers. The framework results were 0.91, 0.74, 0.93, and 0.63 in terms of AUC, accuracy, specificity, and sensitivity, respectively. The average time spent on the analysis of one X-ray was around 15 s, which does not include the time needed for data transfer.

### Second external validation

The second external validation was conducted using PCCDTT data to confirm that the framework passes the experiment requirements in terms of accuracy and computation time and that the input and output formats are in agreement. Two tests were conducted each consisting of 100 unique X-rays with undisclosed reference diagnoses. The first test resulted in 0.74, 0.68, 0.96, and 0.4 in terms of AUC, accuracy, specificity, and sensitivity, respectively. The results were lower than the required minimal AUC of 0.81. The computation time was of 30 ± 2 s in terms of the average ± standard deviation time needed to process one X-ray. To improve the framework performance, the RSNA database^10^ was added to the training collection of images. Second, the framework architecture was modified to predict not only the presence of an abnormality but also the patient’s gender and age (See “Methods” Section). This modification allowed more accurate analysis of patients with age-related lung changes and women with dense breast tissue or breast implants. The second round of validation using new 100 X-rays resulted in 0.86, 0.86, 0.88, and 0.83 in terms of AUC, accuracy, specificity, and sensitivity, respectively. The computation time was of 38 ± 7 s. The databases were sampled from the same hospitals where the experiment was conducted. The aim of this validation was not only to test the performance of the framework but mostly to be sure that the framework can correctly read and process the input X-rays within the required time limits, and that the framework output format is acceptable. The changes in computation time between the first and second external validations are due to the fact that the first validation run on a local machine and measured only the time needed to process an X-ray image, while the second validation measured the total time between sending images from Moscow servers and receiving the report with diagnostic heatmap. The heatmaps were required so that a committee of radiologists from the experiment organizers can perform in-depth inspections of the framework performance on randomly selected collections of X-rays.

### Patient population

In total, 17,888 out of 20,494 X-rays have been successfully analyzed by the framework during the experiment month from 11.23.2020 to 12.25.2020. The 2,458 X-rays have not been analyzed due to technical issues with data transferring. The analysis of 148 X-rays was unsuccessful due to the execution time exceeding the permitted maximum of 6.5 min. The framework rejected 173 X-rays by considering them to either not represent a frontal chest X-ray or be corrupted or cropped. The participating hospitals provided diagnoses for 11,902 X-rays, which allows for the quantitative evaluation of the framework performance. The 11,902 X-rays imaged 11,094 distinct patients, meaning that some patients were images multiple times during treatment. likely. The maximal number of X-rays for a patient was eight. For consistency, the secondary X-rays for such patients were in the database summary analysis. This annotated X-ray collection consisted of 6826 females, 5068 males, and 8 with gender not reported. The patient's ages ranged from 18 years old to 100 years old with a median age of 53 years old. Among all patients, 6818 were under inpatient care, whereas 5084 patients were under outpatient care. The NLP solution that parsed X-ray reports was trained to detect and extract 24 labels of interest. These labels included 17 clinical findings potentially associated with thoracic diseases, namely pleural effusion, infiltrate, dissemination, cyst, calcification, pulmonary mass, focal pulmonary opacity, atelectasis, pneumothorax, pneumonia pocket, tuberculosis, pneumoperitoneum, fibrosis, herniation, cardiomegaly, widened mediastinum, and hilar enlargement. Two labels indicated the presence of musculoskeletal diseases, namely rib fractures, and scoliosis. One label indicated if the imaged patient has a consolidated bone fracture. One label was used to record the cardiothoracic ratio for patients with cardiomegaly. One label indicated if the patient has lung aging changes, which are not associated with a particular disease. Finally, two labels were used to record if the attending radiologist suggests acquiring a new chest X-ray or CT image, respectively. The summary of the database is given in Table 1.

**Table 1:** The summary of the experiment database with the reference abnormality reports.

Characteristic	Value
Chest X-ray	11,902
Unique patients	11,096
**Care type**	
Inpatient	5084
Outpatient	6818
**Demographics**	
Gender, female	6826
Age, median and range	53 [18–100]
**Chest abnormalities**	
Effusion	365
Infiltrate	2477
Pleural dissemination	8
Cyst	10
Pulmonary mass	2086
Opacity	2304
Atelectasis	64
Pneumothorax	95
Pneumonia	873
Tuberculosis	108
Pneumoperitoneum	2
Fibrosis	711
Hernia	23
Cardiomegaly	1919
Widened mediastinum	42
Hilar enlargement	38
**Other abnormalities**	
Scoliosis	180
Bone fracture	78
Consolidated bone fracture	104
Patients with no abnormalities	7872
Patients with multiple abnormalities	3078
Patients with lung aging changes	1523
X-ray retake recommended	12
CT recommended	6

### Experiment results

**Figure 1:**
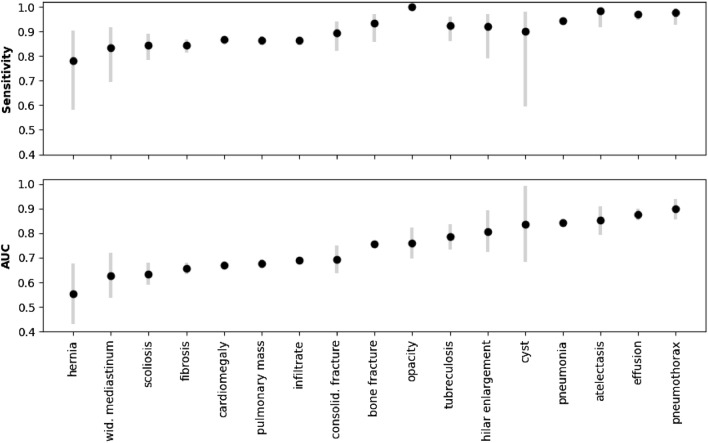
The results of proposed framework on the experiment database. The results are presented in terms of the sensitivity and area under the receiving operator curve (AUC) with 95% confidence intervals. The abnormalities are sorted according to the average value of the AUC. The recognition of consolidated bone fractures has not been counted towards the overall algorithm performance.

The experiment performance was evaluated using AUC, accuracy, specificity, and sensitivity measurements. The AUC value was 0.77, specificity—0.56, and sensitivity 0.84 for the optimally selected cutoff threshold. We also calculated AUC and sensitivity for individual diseases and against patient subcategories (Fig. 1, Table 2). The positive class was assigned to the patients with the diseases of interest, while the negative class was assigned to the patients without any lung abnormalities. The accuracy and specificity values for individual diseases cannot be computed reliably, as the number of false-positive predictions for all diseases can be significantly higher than the number of positive samples for a particular disease. It is also important to mention that the use of binary prediction labels boosts the metric for individual diseases. Indeed, the framework does capture errors when an X-ray with one disease is automatically labeled to have another disease. The results were computed for patient subgroups to quantitatively estimate how the performance changes for patients under inpatient and outpatient care (Table 2) and patients with and without lung aging patterns (Table 3). From the overall results, 79% of cases with multiple abnormalities were correctly classified as abnormal by the framework. The correct classification accuracy drops to 46% for cases with a single abnormality. This accuracy difference is expected assuming that a case with multiple abnormalities is easier to be correctly classified as abnormal.

**Table 2:** The results of proposed framework presented in terms of the area under the receiving operator curve and sensitivity against patients under inpatient and outpatient care.

Outpatient			Inpatient		
Abnormality	AUC	Sensitivity	Abnormality	AUC	Sensitivity
Effusion	0.81 [0.74–0.89]	0.90 [0.78–0.96]	Effusion	**0.87** [0.84–0.90]	**0.98** [0.96–0.99]
Infiltrates	0.66 [0.64–0.68]	0.81 [0.78–0.84]	Infiltrates	**0.69** [0.68–0.71]	**0.89** [0.88–0.91]
Cyst	0.69 [0.35–0.99]	0.67 [0.21–0.94]	Cyst	**0.89** [0.74–0.99]	**1.00** [0.65–1.00]
Pulmonary mass	0.65 [0.62–0.67]	0.81 [0.78–0.84]	Pulmonary mass	**0.68** [0.66–0.70]	**0.89** [0.88–0.91]
Opacity	**0.76** [0.74–0.79]	**1.00** [0.99–0.99]	Opacity	0.74 [0.72–0.76]	1.00 [0.99–1.00]
Atelectasis	**0.86** [0.72–0.99]	**1.00** [0.76–1.00]	Atelectasis	0.83 [0.77–0.90]	0.98 [0.90–1.00]
Pneumothorax	0.80 [0.59–0.99]	1.00 [0.61–1.00]	Pneumothorax	**0.89** [0.85–0.93]	**0.98** [0.92–0.99]
Pneumonia	0.78 [0.75–0.81]	0.88 [0.85–0.91]	Pneumonia	**0.90** [0.88–0.91]	**0.98** [0.97–0.99]
Tuberculosis	0.77 [0.64–0.90]	0.88 [0.66–0.97]	Tuberculosis	**0.77** [0.71–0.82]	**0.93** [0.86–0.97]
Fibrosis	0.65 [0.62–0.69]	0.82 [0.77–0.86]	Fibrosis	**0.66** [0.63–0.69]	**0.86** [0.82–0.89]
Hernia	0.55 [0.37–0.74]	0.70 [0.40–0.89]	Hernia	**0.55** [0.39–0.71]	**0.85** [0.58–0.96]
Cardiomegaly	0.64 [0.61–0.66]	0.81 [0.78–0.85]	Cardiomegaly	**0.66** [0.65–0.68]	**0.89** [0.87–0.90]
Wid. mediastinum	**0.67** [0.47–0.86]	0.78 [0.45–0.94]	Wid. mediastinum	0.59 [0.49–0.69]	**0.85** [0.69–0.94]
Hilar enlargement	0.67 [0.45–0.89]	0.71 [0.36–0.92]	Hilar enlargement	**0.82** [0.73–0.91]	**0.97** [0.84–0.99]
Scoliosis	**0.65** [0.60–0.71]	0.83 [0.74–0.89]	Scoliosis	0.63 [0.57–0.70]	**0.86** [0.77–0.92]
Bone fracture	**0.79** [0.65–0.93]	**1.00** [0.80–1.00]	Bone fracture	0.73 [0.66–0.80]	0.92 [0.83–0.97]
Consolidate fracture	0.67 [0.57–0.78]	0.81 [0.65–0.91]	Consolidate fracture	**0.69** [0.62–0.76]	**0.93** [0.85–0.97]

The higher number for each abnormality is highlighted in bold.

**Table 3:** The results of proposed framework presented in terms of the area under the receiving operator curve and sensitivity against patients with and without sings of lung aging marked by the attending radiologists.

No visual signs of lung aging			Lung aging		
Abnormality	AUC	Sensitivity	Abnormality	AUC	Sensitivity
Effusion	**0.88** [0.85–0.90]	0.96 [0.93–0.98]	Effusion	0.82 [0.77–0.87]	**0.98** [0.94–1.00]
Infiltrates	**0.71** [0.69–0.72]	0.85 [0.83–0.87]	Infiltrates	0.54 [0.51–0.58]	**0.89** [0.87–0.90]
Cyst	0.80 [0.62–0.99]	0.88 [0.53–0.98]	Cyst	**0.96** [0.76–1.00]	**1.00** [0.34–1.00]
Pulmonary mass	**0.69** [0.67–0.71]	0.84 [0.82–0.87]	Pulmonary mass	0.54 [0.50–0.57]	**0.89** [0.87–0.91]
Opacity	**0.78** [0.76–0.80]	**1.00** [1.00–1.00]	Opacity	0.60 [0.57–0.64]	1.00 [1.00–1.00]
Atelectasis	**0.87** [0.80–0.94]	0.98 [0.87–1.00]	Atelectasis	0.74 [0.62–0.85]	**1.00** [0.86–1.00]
Pneumothorax	**0.90** [0.85–0.94]	0.98 [0.92–0.99]	Pneumothorax	0.90 [0.78–1.00]	**1.00** [0.70–1.00]
Pneumonia	**0.84** [0.82–0.86]	0.94 [0.92–0.96]	Pneumonia	0.79 [0.74–0.84]	**0.96** [0.92–0.98]
Tuberculosis	**0.79** [0.73–0.84]	0.91 [0.83–0.96]	Tuberculosis	0.70 [0.58–0.82]	**0.96** [0.81–0.99]
Fibrosis	**0.64** [0.61–0.67]	0.81 [0.77–0.84]	Fibrosis	0.56 [0.50–0.61]	**0.90** [0.85–0.93]
Hernia	**0.53** [0.39–0.67]	0.75 [0.51–0.90]	Hernia	0.48 [0.27–0.69]	**0.86** [0.49–0.97]
Cardiomegaly	**0.67** [0.65–0.69]	0.84 [0.82–0.87]	Cardiomegaly	0.51 [0.47–0.56]	**0.89** [0.87–0.90]
Wid. mediastinum	**0.72** [0.39–1.00]	**1.00** [0.44–1.00]	Wid. mediastinum	0.48 [0.39–0.58]	0.82 [0.67–0.91]
Hilar enlargement	**0.81** [0.71–0.91]	**0.96** [0.81–0.99]	Hilar enlargement	0.74 [0.57–0.90]	0.83 [0.55–0.95]
Scoliosis	**0.62** [0.56–0.67]	0.82 [0.74–0.87]	Scoliosis	0.58 [0.49–0.68]	**0.93** [0.82–0.98]
Bone fracture	**0.76** [0.68–0.83]	0.93 [0.83–0.97]	Bone fracture	0.67 [0.54–0.79]	**0.96** [0.79–0.99]
Consolidate fracture	0.65 [0.57–0.72]	0.83 [0.73–0.90]	Consolidate fracture	**0.68** [0.59–0.78]	**1.00** [0.91–1.00]

The higher number for each abnormality is highlighted in bold.

Every week of the experiment month, 20 random X-rays with the framework results have been manually inspected by a committee of physicians from PCCDTT in order to quantitatively assess the performance. This assessment first checked the completeness of the framework reports by ensuring that they include the original image with the abnormality heatmap and whether the framework correctly labeled the X-ray as pathological or normal. This visual inspection also checked if the abnormality heatmaps are in agreement with the actual pathology manifestation. Finally, the inspection can resolve other uncertainties. Out of 100 X-rays with framework results manually inspected by the committee, 83 were considered acceptable by the committee.

### Experiment result statistics

The experiment was public with monetary awards for the successful participants and therefore attracted attention from companies focused on the use of AI in medicine. In total, 16 commercial companies and one non-commercial research institution participated. It was not required for the participants to reveal their algorithms and publicly share their results due to potential commercial interests. Considering these restrictions, we decided to summarize the statistics of the participants’ performance to assess the experiment challenges. All successful participants passed the external validations (see “Second external validation” section). The AUC for the external validations ranged from 0.5 to 0.94, while the average AUC was 0.88 ± 0.05. At the end of the experiment, the average AUC, sensitivity, and specificity were 0.75 ± 0.02, 0.65 ± 0.11, and 0.74 ± 0.09. The execution time ranged from 12 to 1138 s with a median time of 22 s. In total, the analysis of 21.3% of X-rays exceeded the permitted maximum of 6.5 min. It must be noted that the presented accuracy metrics do not include cases when an algorithm fails to generate a report for an X-ray due to technical errors. For all participating algorithms, the average rate of technical errors was around 5% with a 95% confidence interval of [3.4; 6.6].

## Discussion

The advances of AI in the last ten years have revolutionized many scientific areas, where large quantities of data need to be analyzed. Computer vision and medical image analysis are the fields that benefited significantly from the AI revolution, and human-level performance is commonly reported for various tasks in these fields. One of the problems of such studies is that algorithms are often tested on the internal datasets sampled from the same source as training images, which makes the results subjected to dataset bias, potential data contamination, and low data representativeness. To mitigate these issues, large-scale multicenter studies have been conducted allowing the researchers to better estimate the prospects of AI for, for example, eye disease diagnosis^11,12^. Such studies are, however, expensive and lengthy. As an alternative to multicenter studies, public medical imaging competitions are commonly accepted as one of the most reliable ways for AI algorithm validation in CAD^13,14^. In this paper, we reported the public megapolis-level experiment testing the performance of AI for chest X-ray analysis in 178 clinical centers.

**Figure 2:**
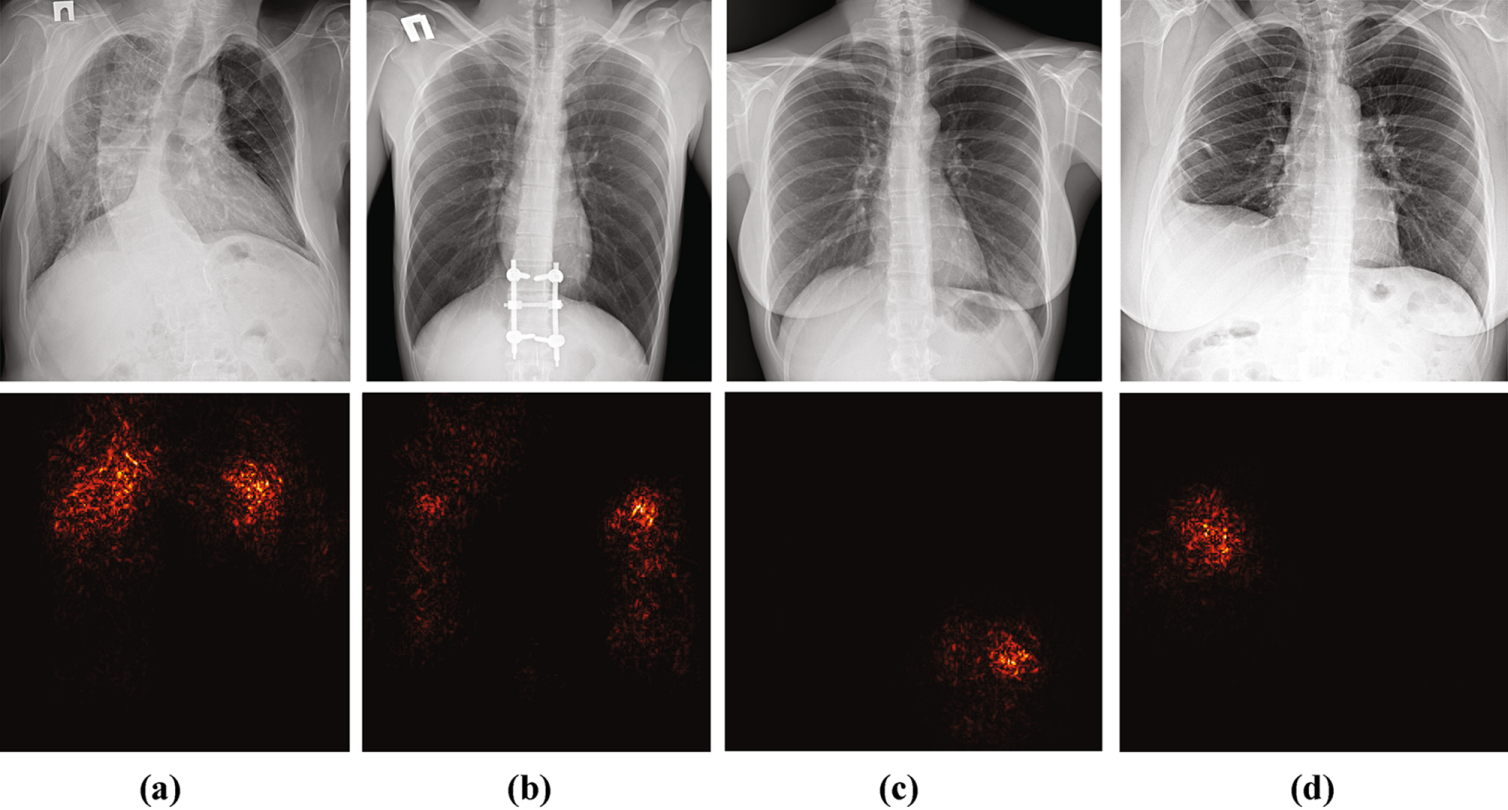
A physician has visually reviewed cases with the framework results. This Figure presents some potentially challenging examples of chest X-rays labeled as abnormal by the framework with the corresponding abnormality heatmaps. (**a**) This case has been manually labeled as pathological requiring a CT image for specific abnormality confirmation. (**b**) An X-ray with no visual abnormalities. According to the physician’s opinion, the framework mis-labeled this X-ray as abnormal due to a wrong patient position so that his shoulder blades overlapped with the lung tissue. (**c**) The physician confirmed the presence of infiltration in the location marked by the framework. (**d**) The algorithm correctly labeled the X-ray by capturing fibrosis but did not highlight pleural effusion and broken ribs.

In the following subsections, we summarized the existing work and the reported results for different abnormalities and present them in comparison to our performance. For the framework deployment, we had to define the decision boundary threshold of the diagnostic neural network. The threshold changes will increase the framework sensitivity at the cost of reduced specificity and vice versa. We aimed at increasing sensitivity as higher sensitivity will result in fewer sick patients being labeled as healthy. Simultaneously, more healthy patients were labeled as sick potentially increasing the radiologists’ workload. Favoring sensitivity seems a reasonable decision as the framework was deployed for both inpatient and outpatient clinical centers. When deploying the framework at different centers, the decision boundary threshold can be adjusted depending on the expected proportion of sick and healthy patients. Due to the experiment execution protocol and testing data restriction, only an approximate performance comparison against existing solutions is possible. We must acknowledge that the results in terms of correctly labeled pathological X-rays are an overestimation of the true positive rate. At the same time, our results have been obtained in the most challenging close-to-real-life settings when the algorithm development team had no access to testing X-rays, and the list of potential abnormalities of interest (Fig. 2). As it was reported by Cohen et al.^6^, using testing data from a different hospital than training data can result in more than 50% reduction of the automated diagnosis accuracy.

### Pulmonary infiltrates

Pulmonary infiltration is the most prevalent abnormality diagnosed by the physicians on the X-rays of the experiment. The infiltrate is a broad and often non-specific term that encompasses visual abnormalities in chest X-rays that edema, blood, exudate, and cancerous tissues^15^. Due to its broad definition, infiltration is the most prevalent class in one of the most popular public lung X-ray databases – ChestX-ray8 from the National Institutes of Health (NIH)^16^. The availability of public data has summoned significant attention from the data science community to the automated infiltrate detection problem^6,17–34^. Most of the authors addressed the problem using classification neural networks usually with ResNet^16,29,31,33,34^ and DenseNet^6,20,28,32,34^ backbones. The performance of the published solutions tested on the NIH database mostly lies in a narrow interval from 0.689 to 0.727 AUC^17,20,21,23–26,28,30,31,34,35^. Augmenting a classification network with lung field segmentation^28^ or abnormality region enhancement^22,23^ resulted in a 1–2% of accuracy improvement. Baltrusch et al.^30^ have not observed a significant improvement in the infiltration detection accuracy from the automated rib suppression. To the best of our knowledge, Cohen et al.^6^ was the only group to use different external testing databases for the infiltration detection evaluation and observed that the accuracy drops to 0.51 AUC in comparison to 0.75 AUC achieved when the testing database is sampled from the same source as the training database. Our framework achieved the AUC of 0.691 and correctly labeled as pathological 87% of X-rays containing infiltrations.

### Pulmonary opacities

Pulmonary opacities were the second-largest abnormality class in the experiment accounting for 2304 abnormality labels. Similar to pulmonary infiltrates, pulmonary opacity is a relatively general term that encompasses various diseases including pulmonary infections, edemas, and cancer. The lung opacities are included in the public Stanford CheXpert database, which significantly stimulates the research of automated opacity diagnosis^36^. The reported results on opacity detection range from 0.90 to 0.94 AUC when training and testing X-rays were sampled from the same public or private databases^6,37–43^. DenseNet^6,37–39^, ResNet^37,39,40^ and Xception^37,39,43^ are the main network architectures used for opacity detection. Considering that opacity is strongly connected with other lung abnormalities, Chakravarty et al.^37^ have demonstrated that graph-based networks that capture inter-disease relationships can slightly improve opacity detection. Testing on external databases dramatically reduced the AUC from 0.93 to 0.74 for the same DenseNet-based model^6^. Lenga et al.^38^ demonstrated that the inclusion of 5% of the external database for training will increase the AUC to 0.77 AUC. The proposed framework achieved the AUC of 0.760 and correctly labeled as pathological 86% of X-rays containing opacities.

### Pulmonary mass

Pulmonary masses, defined as pulmonary opacities larger than 30 mm, are present in 2086 X-rays in the experiment database. Lung masses are included in the NIH database and their automated detection accuracy is currently estimated to be from 0.79 to 0.87 AUC^6,17–21,23–29,31,33,35^. Two studies used private X-ray datasets and were able to demonstrate the performance way above the results of other groups on the public NIH database with the AUC of 0.95–0.97^22,41^. Both papers stated that their neural networks surpassed the inter-observer performance by a significant margin. It is, however, important to indicate that these two databases have a relatively low number of pulmonary mass cases with as low as 70 X-rays with masses in the testing dataset^41^. In contrast, the Google investigation obtained a significantly worse performance of 0.72 AUC on a private database of around 650 k X-rays, which was inferior to the performance on the NIH database^43^. External validation comparison has resulted in a drop from 0.94 AUC, when testing X-rays are sampled from the same database, to 0.638 AUC when testing X-rays are obtained from separate data sources^6^. The DenseNet has been selected by a majority of papers that relied on the existing neural network architectures^6,18–20,26,28,32,44^. The proposed framework achieved the AUC of 0.678 and correctly labeled as pathological 87% of X-rays containing pulmonary masses.

### Cardiomegaly

Heart enlargement, i.e. cardiomegaly, has been present on 1919 X-rays in the experiment database. In contrast to many other lung abnormalities manifested on X-rays, cardiomegaly is formally diagnosed through morphometric analysis of the heart and lung fields, which potentially leaves less room for subjective decisions and human errors. Moreover, machine learning algorithms can be trained to segment lungs and heart from X-rays and then derive the cardiac measurements from the resulting segmentation. The existing papers that follow the diagnosis-from-segmentation approach have demonstrated a very high performance with AUC from 0.935 to 0.977 using internal validation^45–48^. In all these papers, the UNet network was used for heart and lung segmentation. The standard end-to-end solutions, where networks are asked to predict the disease by directly analyzing the raw X-ray, resulted in AUC from 0.60 to 0.89 tested on the NIH database^17,18,20,23–29,31,33,34^. The results on alternative public databases such as CheXpert, MIMIC, OpenI, and PadChest, and their combination with private databases can reach 0.945 AUC^19,21,22,30,32,35–37,39,42,44,49–51^. There is no agreement on whether the richness and representativeness of public databases are sufficient to develop cardiomegaly detection algorithms with comparable performance on internal and external data sources. Three studies have demonstrated the external and internal validation resulting in similar AUC values from 0.800 to 0.828^38,52,53^, while Cohen et al.^6^ observed a drastic drop from 0.945 AUC to 0.721 AUC. Rajpurkar et al.^32^ observed that cardiomegaly is one of the lung abnormalities where automated detection results are significantly lower than the inter-observer variability. The proposed framework achieved the AUC of 0.671 and correctly labeled as pathological a 73% of X-rays with cardiomegaly, which is lower than the detection rate of other abnormalities. Considering the proportion of positive and negative predictions of the presented framework, the results for cardiomegaly are not significantly better than random guessing.

### Pneumonia

Pneumonia accounts for 873 X-rays in the experiment database. In 2018, the Radiological Society of North America organized a public competition on automated pneumonia diagnosis from chest X-rays with monetary awards, which considerably stimulated the interest in the topic from the research society and allows us to objectively estimate the performance of the algorithms when the testing data is sampled from the same source as training but not available to the algorithm developers. The Dice coefficient for pneumonia pockets localization was 0.29 for one of the top-performing teams who published their results^54^. During the later reuse of the training part of the database, the researchers reported an AUC of 0.74–0.85 and intersection-over-union of 0.54 for pocket localization^55,56^. The AUC values of 0.69–0.74 are obtained on other public databases such as NIH and CheXpert^20,23,24,37–39^. In contrast to the agreement observed on public databases, the results on private databases vary significantly and can reach 0.95 AUC^57^. Two studies that compared internal and external validation of automated pneumonia detection have reported a significant performance reduction for the external validation with the accuracy dropping from 0.68 to 0.47^58^, and AUC from 0.90 to 0.59^6^. The recent outbreak of COVID-19 disease has given an additional impetus to automated pneumonia diagnosis, especially to the recognition of various pneumonia types. The accuracy of COVID-19 diagnosis against healthy X-rays is around 0.76–0.90 AUC^57,59–62^, whereas the differentiation between COVID-19 and non-COVID-19 pneumonia reaches the accuracy of 0.92 AUC^63,64^. The proposed framework achieved the AUC of 0.842 and correctly labeled as pathological a 95% of X-rays with pneumonia. Hu et al. have observed that the use of dual-energy X-ray images with ribs suppressed does not significantly improve pneumonia detection accuracy^60^.

### Pneumothorax

Pneumothorax has been reported for 95 X-rays in the experiment database. Although pneumothorax is not as common as the previously discussed lung diseases, it could be life-threatening without urgent attention and therefore receives considerable attention from the medical imaging research community. The Society for Imaging Informatics in Medicine (SIIM), the American College of Radiology (ACR), and the Society of Thoracic Radiology (STR) jointly organized a public competition on automated pneumothorax diagnosis from X-rays. The best submitted AI solution segmented pneumothorax pockets with 0.87 Dice^65^. It is important to note that the Dice values were slightly biased by the fact that the number of healthy X-rays in the SIIM competition database is relatively high, and the correct recognition of healthy X-rays results in Dice of 1.0, which boosts the average Dice score. The comparison of AI to three radiologists on challenging-to-analyze cases from SIIM has demonstrated that AI can segment pneumothorax pockets more accurately than the radiologists, while the radiologists were more accurate in pneumothorax/no pneumothorax classification^66^. The results on the NIH database are around 0.80–0.98 AUC^17,19,20,23^. The superior results on the SIIM challenge, where the testing labels are not available to the algorithm developers, in contrast to the result on the NIH database suggest that binary pneumothorax diagnosis could be simpler than pneumothorax diagnosis as a part of a multi-disease analysis. Existing reports on external validation of pneumothorax diagnosis have demonstrated a drop in accuracy from 0.92 to 0.463^6^, and from 0.90 to 0.59^7^. The proposed framework achieved the AUC of 0.898 and correctly labeled as pathological a 98% of X-rays with pneumothorax pockets.

### Binary classification

Although most of the existing papers perform multi-disease analysis due to the availability of public annotated databases, some recent papers focused on binary lung disease classification. One of the key ideas investigated was increasing the number of true negative predictions while keeping the number of false negatives as low as possible, i.e. focusing on the recognition of healthy cases. Dyer et al.^67^ tested how well DenseNet can recognize X-rays from healthy subjects, and observed that it mislabels 4% of abnormalities for 20% of X-rays labeled with the highest probability of healthiness. Wong et al.^68^ separated X-rays into easy, where three radiologists gave the same diagnosis, and challenging, where only two radiologists agreed on the diagnosis. Using easy cases, their algorithm recognized 33% of healthy X-rays without missing an abnormality. This number dropped to 23% for challenging cases. In the proposed study, the framework mislabels 4% of abnormalities against a 16.5% true negative rate for 13% of X-rays labeled with the highest probability of healthiness.

The chest X-rays were acquired for patients both under inpatient and outpatient, i.e. ambulatory, care. As ambulatory patients include a large number of patients that underwent routine or pre-employment X-ray screening, a framework that can handle ambulatory X-rays can potentially benefit more patients. Moreover, early signs of diseases are more likely to be missed during the screening of ambulatory patients, where healthy X-rays significantly prevail over pathological X-rays. The X-rays from inpatient care centers are from patients who cannot be treated at home and require hospitalization, so we can expect a higher prevalence of lung abnormalities in such chest X-rays. An expected proportion of healthy vs. abnormal cases for the analysis will affect the optimal framework parameters such as the classification boundary. The training databases included both inpatient and ambulatory care cases. The X-rays from City Hospital #7, and City Hospital #18 were mainly from routine scanning with 97% and 98% healthy subject prevalence, respectively. The X-rays from Republic TB Dispensary were composed of inpatient and outpatient care patients with 78% healthy subject prevalence. The public database used for training did not provide information on the type of care for their patients except for CheXpert, where the authors mentioned that X-rays from both inpatient and outpatient centers were used. We could, however, assume that other databases also had X-rays from inpatient centers as some patients are imaged in the horizontal position using portable X-ray machines. The testing database had 57% and 43% of outpatient and inpatient cases, respectively. For most of the abnormalities, the framework performance was superior for outpatient cases (Table 2). We asked radiologists with 3-year and 30-year experience in chest X-ray image analysis to retrospectively inspect some of the outpatient and inpatient X-rays and comment on the network performance and visual diagnostic uncertainties. They observed that the nodules and potential tuberculosis cavities in the ambulatory are relatively small and are likely to be missed by the framework and even by some radiologists. The infiltrates in lung basal segments are likely to be missed in ambulatory patients with unspecific clinical presentations. Pneumothorax accompanies various lung diseases or could be the result of lung tissue biopsy, which considerably increases the occurrence of pneumothorax for patients under inpatient care. Outpatient patients with compensated heart failure may have small pleural effusion pockets, which are more difficult to automatically detect in contrast to ambulatory patients with decompensated heart failure, where lungs pleural effusion pockets are larger and better visible. Both radiologists agreed that improving the framework performance will require the use of additional data sources such as clinical reports and patient disease history.

The patient’s age is usually of diagnostic importance, and therefore several attempts for its automated estimation with AI have been performed^69,70^. Aging significantly affects human lungs and their appearance in X-ray images. Certain visual patterns manifested in young patients are more likely to be associated with lung diseases than the same patterns manifested in elderly patients. One of the reasons is that the accumulated risks of having lung diseases grow with time so elderly patients are more likely to have signs of previously experienced diseases in their lung fields. For example, visual consolidations in lung basal segments are often associated with congestive heart failure in elderly patients. Osteoarthritis of the sternoclavicular joint could obstruct potential abnormalities in X-rays or be identified as a false positive abnormality. The attending physicians indicated which patients had visual signs of lung aging in their reports. The framework was trained to predict the age from X-ray. To understand if lung aging perfectly correlates with age on the experiment database, a logistic regressor was trained on age features to predict lung aging labels. The regressor performance was 0.79 and 0.70 in terms of AUC and prediction accuracy, respectively. The optimal cut-off threshold was 60.1 years which qualified all patients with lung aging signs into the correctly classified patient category. The experiment cases were subdivided into patients with/without lung aging, and patients, for whom the logistic regressor correctly/incorrectly predicted lung aging. The results for the two subdivisions are presented in Tables 3 and 4. There was no significant difference between the framework results computed for patients without lung aging and patients whose lung aging is in agreement with their age according to the logistic regressor. Similarly, there was no significant difference between the framework results computed for patients with lung aging and patients whose lung aging was not in agreement with their age according to the logistic regressor. This observation leads to a non-obvious conclusion on the efficient analysis of age-related information. It seems to be insufficient to simply use the age feature or train the framework to recognize age-related changes in X-rays. The framework needs to be trained to recognize cases where the patient’s age is not in agreement with age-related lung changes. To the best of our knowledge, there is only one paper that estimated the age from chest X-rays, which observed an average error of 4.7 and 4.9 years using DenseNet121 and ResNet50 networks, respectively^71^.

**Table 4:** The age information is integrated into the proposed framework.

Visual lung aging match age			Visual lung aging does not match age		
Abnormality	AUC	Sensitivity	Abnormality	AUC	Sensitivity
Effusion	**0.89** [0.86–0.92]	0.97 [0.94–0.99]	Effusion	0.84 [0.80–0.88]	**0.97** [0.93–0.99]
Infiltrates	**0.70** [0.68–0.71]	0.86 [0.84–0.88]	Infiltrates	0.66 [0.64–0.68]	**0.87** [0.85–0.89]
Cyst	**0.87** [0.72–1.00]	0.88 [0.53–0.98]	Cyst	0.64 [0.23–1.00]	**1.00** [0.34–1.00]
Pulmonary mass	**0.70** [0.68–0.71]	**0.87** [0.85–0.89]	Pulmonary mass	0.63 [0.60–0.65]	0.86 [0.83–0.88]
Opacity	**0.77** [0.75–0.78]	**1.00** [1.00–1.00]	Opacity	0.72 [0.70–0.74]	1.00 [1.00–1.00]
Atelectasis	0.83 [0.74–0.93]	0.96 [0.82–0.99]	Atelectasis	**0.85** [0.77–0.93]	**1.00** [0.91–1.00]
Pneumothorax	0.87 [0.81–0.93]	**0.98** [0.92–0.99]	Pneumothorax	**0.93** [0.88–0.98]	0.98 [0.88–1.00]
Pneumonia	0.81 [0.79–0.84]	0.92 [0.89–0.94]	Pneumonia	**0.87** [0.85–0.89]	**0.98** [0.96–0.99]
Tuberculosis	**0.79** [0.73–0.85]	0.91 [0.83–0.96]	Tuberculosis	0.77 [0.67–0.88]	**0.96** [0.82–0.99]
Fibrosis	**0.71** [0.68–0.74]	**0.89** [0.85–0.91]	Fibrosis	0.55 [0.51–0.58]	0.79 [0.74–0.83]
Hernia	**0.58** [0.43–0.74]	**0.79** [0.52–0.92]	Hernia	0.46 [0.28–0.65]	0.78 [0.45–0.94]
Cardiomegaly	**0.70** [0.68–0.72]	**0.88** [0.86–0.90]	Cardiomegaly	0.60 [0.57–0.62]	0.85 [0.82–0.90]
Wid. mediastinum	**0.74** [0.64–0.85]	**0.93** [0.77–0.98]	Wid. mediastinum	0.35 [0.22–0.48]	0.64 [0.39–0.84]
Hilar enlargement	**0.87** [0.76–0.97]	**1.00** [0.83–1.00]	Hilar enlargement	0.72 [0.58–0.85]	0.84 [0.62–0.94]
Scoliosis	**0.65** [0.60–0.70]	0.83 [0.76–0.86]	Scoliosis	0.60 [0.52–0.68]	**0.88** [0.76–0.94]
Bone fracture	**0.77** [0.70–0.85]	0.92 [0.82–0.97]	Bone fracture	0.73 [0.62–0.84]	**0.96** [0.81–0.99]
Consolidate fracture	0.66 [0.58–0.73]	0.83 [0.71–0.90]	Consolidate fracture	**0.74** [0.65–0.82]	**1.00** [0.91–1.00]

A separate logistic regression model was implemented to predict visual lung aging from the age feature. The model performance was of 0.79 and 0.70 AUC and accuracy. This table presents the framework results in terms of the area under the receiving operator curve and sensitivity against the patients whose visual lung aging was correctly/incorrectly predicted from their age. The higher number for each abnormality is highlighted in bold.

## Methods

A framework for automated chest X-ray analysis has been developed. Considering that the framework was deployed for everyday clinical practice, it included components for input data validation and preprocessing. In particular, one module of the framework checked whether an input image represents a conventional chest X-ray without grayscale inversion. Another module was trained to detect lung fields in chest X-rays. Such a modular structure facilitates the upgrading of individual framework parts without the need for complete framework retraining and simplifies framework validation.

### Training databases

To facilitate the generalization capability of the proposed framework, it was trained on a rich collection of public and private X-ray images. Three public databases, namely ChestX-ray8 (NIH)^16^, CheXpert^36^, and RSNA^10^ databases were used for training. After combining these public databases, we formed a training database with 35,115 X-rays with atelectasis, 24,102 with cardiomegaly, 82,027 with pleural effusion, 10,317 with infiltration, 6046 with pulmonary masses, 12,643 with nodules/lesions,13,910 with pneumonia, 20,106 with pneumothorax, 92,669 with opacities, 48,905 with edema, 12,730 with consolidation and 7270 with bone fractures. Note that one X-ray may have multiple abnormalities present. The private databases were obtained from City Hospital #7, City Hospital #18, and Tuberculosis Dispensary from the city of Kazan, Russia. The collection of 59,944 private X-rays included the same abnormalities as public databases plus tuberculosis. Prior to the framework training, the labels of the X-rays were converted to binary, i.e. all abnormality labels were united into a single class. In summary, the X-rays for training were collected from different sources, acquired using different imaging equipment, imaged patients in both vertical and horizontal positioning, and had different spatial resolutions.

### Training data augmentation

To enrich the training databases, the X-rays were augmented with intensity and geometry transformations during training. The intensity augmentations included: brightness augmentation with the factor of 0.2; contrast augmentation of 0–5 percent magnitude; gamma augmentation of level [70; 130]. For each training image, a random intensity augmentation with random parameters was selected. With 0.5 probability, one additional intensity augmentation was selected between additive Gaussian noise with the variance [10; 50] or blur with the maximal standard deviation of 5. The geometry transformations included random X-ray rotations of up to 20 degrees and image scaling by the maximum factors of 0.15. A random combination of rotation and scaling was applied to each training image. With 0.5 probability, a training image may be flipped in the horizontal direction. Such training data augmentation can improve diagnostic accuracy by 2–4%^72^.

### Input image preprocessing

**Figure 3:**
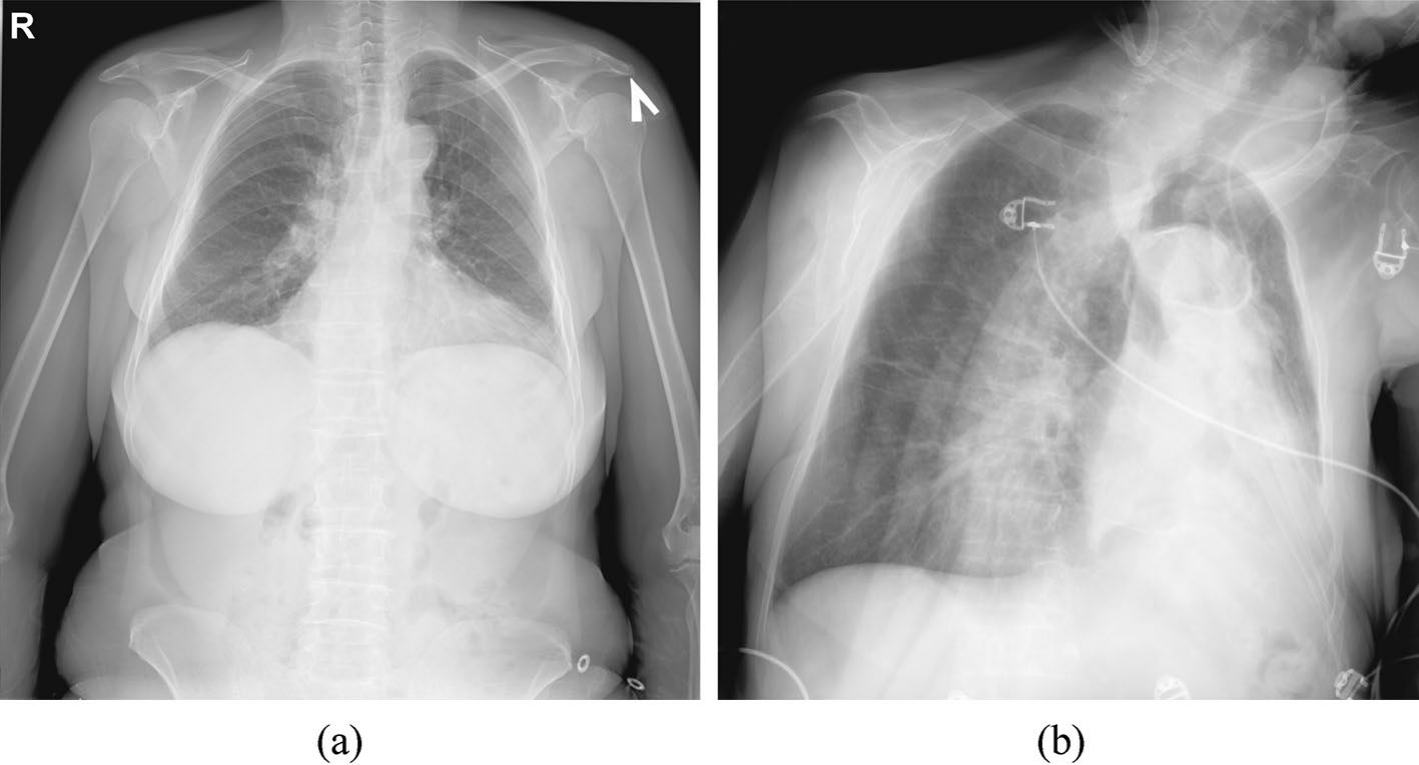
(**a**) A chest X-ray example, where the inclusion of gender prediction head of the framework corrected a false-positive disease prediction. (**b**) A chest X-ray example mis-classified as defected, i.e. not representing a fronal chest X-ray.

Before being analyzed for chest abnormalities, an input image passed through several preprocessing steps. First, a neural network scanned the image to recognize if it represents a conventional or grayscale-inverted X-ray. An X-ray marked as grayscale-inverted was then converted to conventional. Second, a neural network scanned the image to recognize if it represents a frontal chest X-ray or lateral chest X-ray, or some other image. An input not labeled as a frontal chest X-ray is marked as defected and no further analysis is performed on it. The EfficientNet classification network architecture was used for both preprocessing steps. The networks were trained with a combination of binary cross-entropy and focal losses, Adam optimizer, and l2 regularization with a weighting factor of 0.0001. In the third preprocessing step the approximate location of the lung fields was estimated. The X-ray was converted into an integral image to compute Haar, a histogram of oriented gradients (HoG), and local binary pattern features^73^. These features were matched to the lung field descriptors to find the approximate locations of the lung fields in the X-ray. The lung fields are cropped from the input X-ray using the bounding box with safety margins. The lung field detection was needed to normalize the location of the target anatomy, remove unnecessary parts of the head and abdomen that could be present in the X-ray, and recognize defective X-rays where lung fields are cropped. The preprocessing of the input image was needed to automatically recognize if it actually represents a frontal lung X-ray of acceptable quality. It was required by the experiment organizers for frameworks to recognize and report defective inputs. It was considered an error if an automated report is generated for a non-lung X-ray or marked as defective a lung X-ray (Fig. 3b). The cropped lung field region was then rescaled to 512 × 512 size and the intensities were normalized to the [0; 1] range.

### Multi-head diagnostic network

**Figure 4:**
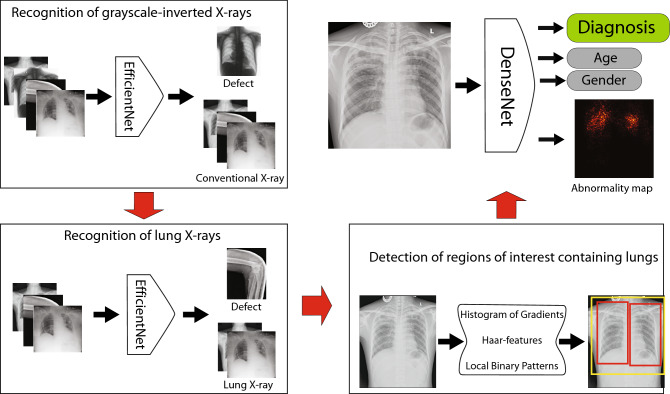
The schematic illustration of the proposed framework consisting of several components for (1) identification of grayscale inverted X-rays, (2) non-lung X-ray images, (3) lung region of interest selection, and automated diagnosis.

A deep multi-head neural network for the prediction of lung abnormalities from chest X-rays was developed (Fig. 4). The encoder part of the network was based on the DenseNet architecture pre-trained on existing chest X-rays databases such as NIH, RSNA, PadChest^74^, CheXpert, and MIMIC^75^ databases. The network was modified to generate multiple outputs of multiple types. A fully-connected layer block with 1024 features and two-value output was added to predict the presence/absence of chest abnormality. A fully-connected layer block with 1024 features and three-value output was added to predict the gender of the patient, i.e. male, female, or other (Fig. 3a). A fully-connected layer block with 1024 features and 10-value output was added to predict the age of the imaged patient. The patient ages were aggregated into bins of 10-year duration, starting from [0; 10] to [90; 100] years. In the first training iteration, all existing network layers were frozen, while the new layers were trained on NIH, RSNA and CheXpert, and private X-rays. In the second training iteration, all network layers were unfrozen and training continued using only private X-rays. For both steps, the training continued until no performance improvement was achieved for 15 consecutive epochs. The training was performed using Adam optimizer with training X-ray separated into 12-image batches. The cross-entropy loss was used for all networks heads (Supplementary Information).

Before the two rounds of external validation, the framework passed several internal validations using randomly sampled parts of the City Hospital #7 and Tuberculosis Dispensary database for validation. Using City Hospital #7 data for validation, the framework performance was 0.84, 0.78, 0.78, and 0.78 in terms of AUC, accuracy, specificity, and sensitivity, respectively. Using Tuberculosis Dispensary data for validation, the framework performance was 0.89, 0.76, 0.63, and 0.89 in terms of AUC, accuracy, specificity, and sensitivity, respectively.

### Ethics

The study has been approved by the institutional review board (IRB) of the Moscow Ministry of Health and Family Welfare Department.

### Informed consent

Informed consent was obtained from all participants. In particular, all the patients were informed that their clinical data will be used for research purposes. All patients were of age, i.e. ≥ 18 years old, so there was no need to get approval from their parents or legal guardians.

### Accordance statement

All methods were performed in accordance with the relevant guidelines and regulations.

## Supplementary Information

Supplementary Information.

## Data Availability

The data from the first or/and second external validation experiment will be made available upon acceptance of the manuscript.
